# RNA Binding Proteins in the miRNA Pathway

**DOI:** 10.3390/ijms17010031

**Published:** 2015-12-26

**Authors:** Patrick Connerty, Alireza Ahadi, Gyorgy Hutvagner

**Affiliations:** 1 Faculty of Science 1, University of Technology, Sydney, NSW 2007, Australia; patrick.connerty@student.uts.edu.au; 2 Faculty of Engineering and Information technology 2, University of Technology, Sydney, NSW 2007, Australia; alireza.ahadi@uts.edu.au

**Keywords:** miRNA, RNA binding protein, Drosha, Dicer, Argonaute, cross-linking and immonoprecipitation (CLIP)

## Abstract

microRNAs (miRNAs) are short ~22 nucleotides (nt) ribonucleic acids which post-transcriptionally regulate gene expression. miRNAs are key regulators of all cellular processes, and the correct expression of miRNAs in an organism is crucial for proper development and cellular function. As a result, the miRNA biogenesis pathway is highly regulated. In this review, we outline the basic steps of miRNA biogenesis and miRNA mediated gene regulation focusing on the role of RNA binding proteins (RBPs). We also describe multiple mechanisms that regulate the canonical miRNA pathway, which depends on a wide range of RBPs. Moreover, we hypothesise that the interaction between miRNA regulation and RBPs is potentially more widespread based on the analysis of available high-throughput datasets.

## 1. Introduction

MicroRNAs (miRNAs) are an abundant class of small regulatory RNAs about 19–22 nucleotides (nt) in length [1]. They have been predicted to regulate the expression of more than 60% of mammalian genes and play fundamental roles in most biological processes including multiple diseases [2,3]. The canonical miRNA pathway starts with the transcription of miRNA genes by RNA polymerase II, which results in the production of the primary miRNA (pri-miRNA). The ~80 base pair long stem loops of the pri-miRNAs are released from the primary transcript generating the precursor miRNA (pre-miRNA). The pre-miRNA itself undergoes multiple processing steps before the mature miRNA is finally generated [4]. Incorporated into one member of the Argonaute (Ago) protein family in the RNA induced silencing complex (RISC), a mature miRNA binds typically to the 3′ untranslated region (UTR) of the targeted messenger RNA (mRNA) [1] and inhibits its translation via various mechanisms [5]. The key determinant of target recognition is a short sequence complementarity between the miRNA seed sequence (the 2nd–8th nucleotides of the miRNA) and the targeted mRNA [6]. The maturation and function of miRNAs are highly dependent on the coordinated action of several RNA-binding proteins (RBP) [7]. Some of these proteins present unique protein domains that are characteristic of proteins involved in small RNA processing and small RNA mediated gene regulatory events [8].

The processing and action of miRNAs are extensively regulated by auxiliary factors to ensure cell/tissue specific functions or adequate response to environmental and cellular stimuli. One of the largest groups of proteins that influence the miRNA pathways are RNA-binding proteins (RBP). The application of advanced biochemical methods such as the use of a variety of cross-linking immunoprecipitations (CLIPs) with the key proteins of the miRNA pathway, accelerated the identification of RBPs that are associated with complexes that are involved in miRNA processing or bind to mRNAs that are targeted by miRNAs [9,10].

In this review, we describe the characteristics and functions of RBPs that are necessary for the production of miRNAs and miRNA mediated gene expression as well as RBPs that regulate these processes in mammalian cells. We have also carried out a basic bioinformatics exercise to compute the potential scale of miRNA and RBP interactions using available CLIP data. Based on this we suggest a more widespread interaction between RBPs and miRNA complexes in the targeting step of miRNA mediated gene regulation.

## 2. RNA Binding Proteins in the miRNA Pathway

### 2.1. Core RNA Binding Proteins of miRNA Processing and miRNA Mediated Gene Regulation

#### 2.1.1. Pri-miRNA Processing by the Microprocessor

The mammalian pri-miRNA contains a double stranded hairpin stem, a terminal loop and two single stranded flanking regions [11]. Recent studies identified additional sequences and structural elements that are necessary for efficient miRNA production. These include the length of the hairpin stem structure, the UGU motif in the apical loop, a GHG motif in the stem, and a UG and CNNC motif in the basal region of the pri-miRNA [12,13,14]. The first step in miRNA biogenesis is the recognition of these motifs and the cleavage of the miRNA hairpins from the primary transcript. This is carried out by the coordinated action of the Microprocessor complex. The Microprocessor is a heterotrimeric complex, which is made up of two proteins: an RNA III enzyme Drosha, and two RNA-binding proteins DGCR8 (DiGeorge Critical Region 8) [12,15,16,17,18].

Drosha (also known as RNASEN) is a Class II RNase III enzyme that characteristically contains two tandem RNase III domains and a dsRNA-binding domain. Drosha is an Mg^2+^ dependent endonuclease that has roles in miRNA biogenesis, ribosomal RNA processing, and viral defence [19].

DGCR8, is the other component of the Microprocessor complex [15,16,17]. It contains a nuclear localization signal, two dsRBDs (double stranded RNA-Binding domains), an RNA-binding heme domain (Rhed), and a C-terminal tail (CTT) [11,20,21,22]. DGCR8 is dimerized through the Rhed domain and binds to Drosha with its C-terminal region [20,23].

**Figure 1:**
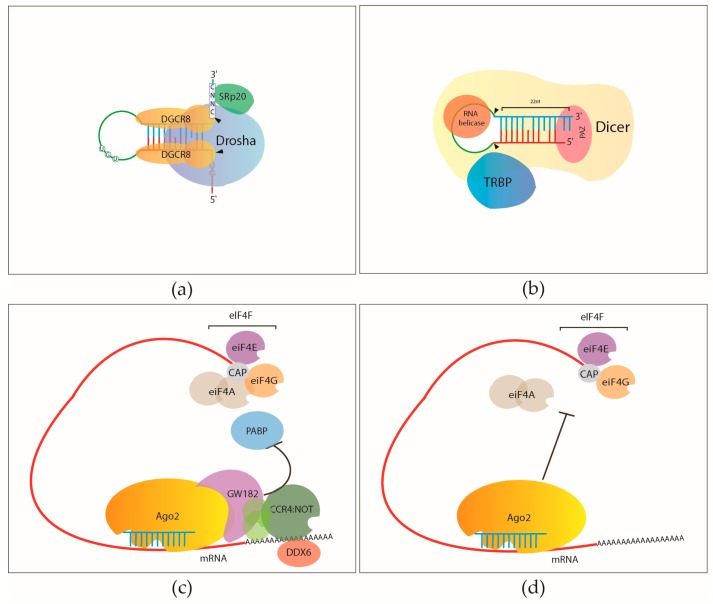
RNA binding proteins of the canonical miRNA biogenesis pathway: (**a**) Sequence motifs and proteins that are required for efficient pri-miRNA processing by the Microprocessor complex; (**b**) Proteins required for pre-miRNA processing; (**c**) Argonaute and GW182 dependent inhibition of translation initiation; and (**d**) miRNA mediated inhibition of translation initiation that is not required GW182.

Recent biochemical data using purified recombinant Drosha and DGCR8 revealed that DGCR8 recognizes the apical UGU motif and binds to the pri-miRNA stem with its dsRBD domains. It also stabilises Drosha with its CTT. In a cleavage competent Microprocessor, Drosha is positioned at the basal UG motif that is at the junction of the single stranded flanking regions and the stem of the hairpin [12]. Drosha cleaves the 5′ and 3′ arFms of the pri-miRNA, 11 base pairs away from the single-stranded flank/double-stranded junction [18]. It was recently demonstrated that a CNNC motif, downstream from the stem-ssRNA junction, is also required for efficient mammalian pri-miRNA processing by the binding of the SRp20 splicing factor through its RNA recognition motif (RRM) [13] (Figure 1a).

Both Drosha and DGCR8 have miRNA-independent functions. Drosha recognizes and cleaves hairpins in mRNAs including the mRNA of DGCR8 that results in an auto regulatory circuitry of the Microprocessor [24,25]. DGCR8 also has RNA-binding functions that are independent from the Microprocessor. For example, a HITS-CLIP (hight-throughput sequencing of RNA isolated from cross-linking immunoprecipitation) screening of RNA targets of DGCR8 in human cells revealed that DGCR8 binds to and mediates cleavage of small nucleolar RNAs (snoRNAs) [10].

#### 2.1.2. Pre-miRNA Processing

After Microprocessor processing, the resulting pre-miRNAs are exported from the nucleus to the cytoplasm via the Exportin 5 pathway [26,27]. In the cytoplasm, pre-miRNAs are further cleaved by Dicer, another RNAse III enzyme, with the help of RNA-binding co-factors which are Protein Kinase, Interferon-Inducible Double Stranded RNA Dependent Activator (PACT) and HIV-1 TAR RNA-binding protein (TRBP) [28,29,30,31].

Dicer is a RNase III enzyme which contains an ATPase/RNA helicase, a PAZ domain (Piwi, Argonaute and Zwille domain is a domain only present in proteins involved in small RNA mediated gene regulation) [8], two catalytic RNase III domains, a domain of unknown function (DUF283) and a C-terminal dsRBD [32]. Dicer was first recognised for cleaving dsRNA into small interfering RNA (siRNA) [33,34] but was later discovered to also process miRNAs [28,29].

The PAZ domain of DICER binds to the 3′ end of the small RNA substrates and also recognizes the 5′ terminal phosphate of an authentic miRNA precursor [35,36,37,38]. The RNA helicase of Dicer recognises the hairpin loop structures of pre-miRNAs and is able to differentiate between pre-miRNA and other double stranded RNAs [39,40,41,42]. Dicer uses the region between its PAZ and RNase III domains as a “molecular ruler” to produce small RNAs with consistent size [43]. Both RNase III domains of Dicer cleaves the pre-miRNA with each RNase domain cleaving one strand of the small RNA duplex [44] (Figure 1b).

Although Dicer is capable of cleaving pre-miRNA and dsRNA alone, its activity is modulated by protein interactors. Two particular proteins well documented in this role are the related PACT and TRBP [30,31]. Both of these Dicer binging proteins have three dsRBDs, one of which facilitates protein-protein interaction by binding to the helicase domain of Dicer [45,46,47]. Both proteins stabilize Dicer [48], affect the fidelity of miRNA processing [47,49,50] and influence the subsequent strand selection of the miRNA [30,47,51].

#### 2.1.3. Argonautes and the RNA Induced Silencing Complex (RISC)

Dicer cleavage produces a short (21–23 nt long) double stranded RNA with a characteristic 2 nt overhang at the 3′ end [52,53]. One of the strands of the miRNA duplex is incorporated into an Argonaute (Ago) protein, forming the minimal effector RNA induced silencing complex (RISC) [54,55,56,57,58,59].

Argonautes are bi-lobed proteins. They consist of an N-terminal , a PAZ domain (similar to the PAZ domain found in Dicer), a middle (MID) domain and a PIWI domain [60,61,62]. The N-terminal domain facilitates small RNA loading and the unwinding of the RNA duplex generated by Dicer [63]. The PAZ domain recognizes and anchors to the 3′ end of the miRNA [64]. The MID domain binds the 5′ terminal monophosphate moiety and the 5′ terminal nucleotide of the miRNA guide strand. The PIWI domain accommodates the miRNA-target RNA duplex, and it folds similarly to RNase H [60].

Ago2 (one of the four mammalian Argonautes) is the only Ago that possesses endonucleolytic activity and can initiate the cleavage of the passenger strand of an extensively base-paired small RNA duplex or the targeted mRNA if it is perfectly complementary to the Ago2 bound small RNA [60,65,66,67,68]. An increasing amount of evidence also suggests that cleavage competent Argonautes also play a role in the processing of a pri-miRNA in *C. elegans* and in the Dicer dependent and independent maturation of pre-miRNAs in mammals and worms [69,70,71,72].

#### 2.1.4. RNA-Binding Proteins Involved in miRNA Mediated Gene Regulation

Since the majority of mammalian miRNAs only share limited complementary to their targets and miRNAs also associate with Argonautes that lack endonuclease activity, target mRNA cleavage is a rare event in mammals. Therefore, miRNAs in mammals mainly inhibit the translation of mRNAs and degrade targeted RNA in a non-sequence specific manner. These processes also involve a range of RNA-binding proteins.

The key proteins of a known mechanism of miRNA mediated translational repression and RNA decay are the members of the TNRC6C/GW182 protein family [73]. They contain an N-terminal, Gly and Trp (GW/WG) repeats in their Argonaute Hook Domain and a RNA recognition motif (RRM) [74]. The TNRC6C protein binds to the PIWI domain of Ago through its GW/WG repeats and localises Ago to the processing bodies (P-bodies) [75]. It also binds to the poly (A)-binding protein (PABP) [76] inhibiting its interaction with the cap binding protein eIF4G that is necessary for translation initiation [77,78]. Ago bound TNRC6Cs also recruit the deadenylase complex CCR4-NOT (Carbon catabolite repression 4-negative on TATA-less) which in turn recruits the DEAD box helicase DDX6 and facilitates the degradation of the miRNA target [76,79,80,81,82] (Figure 1c).

Recent studies in flies and mammalian systems showed that miRNAs could repress translation in a GW182 independent way by inhibiting the formation of the eIF4F complex which recruits the translationally competent mRNA to the ribosomes [83,84,85]. RISCs facilitate the release of ATP-dependent RNA helicase proteins (eIF4A) from the eIF4F complex and therefore inhibit translation of the miRNA targeted mRNA [84,85] (Figure 1d).

### 2.2. RNA-Binding Proteins that Regulate miRNA Biosynthesis

**Figure 2:**
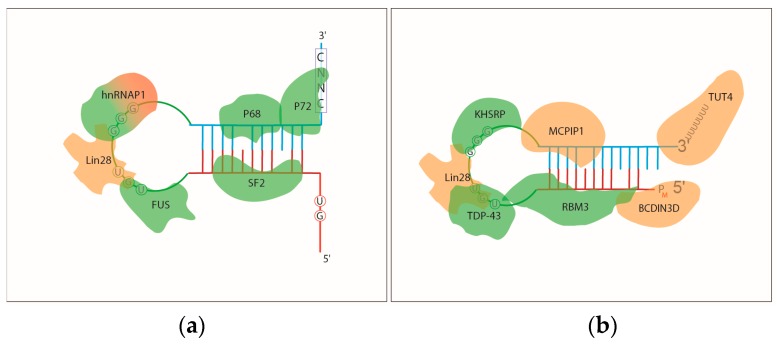

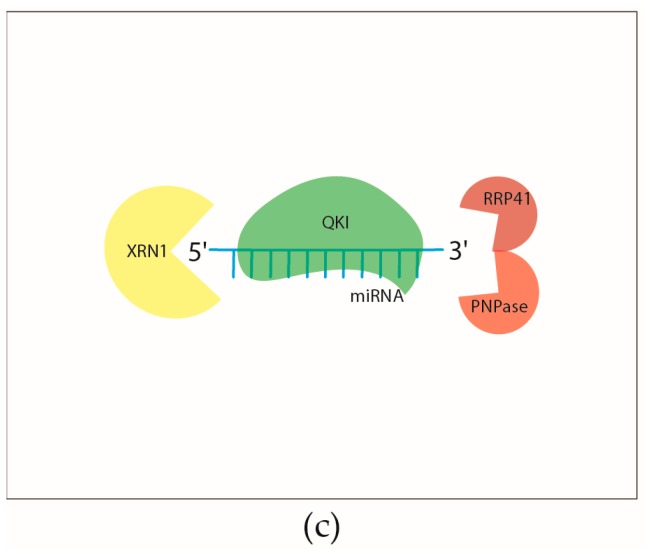
RNA binding proteins that regulate (**a**) pri-miRNAs and (**b**) pre-miRNAs biogenesis by recognizing sequences or structures on the hairpin RNA. RBPs labeled with green are promoting miRNA processing and PBPs coloured with orange are inhibitors of the miRNA pathway; (**c**) RBPs that regulate the stability and turnover of mature miRNAs.

miRNA processing is regulated at each step of the maturation pathway by multiple RBPs to ensure tissue specific expression or proper response to environmental and cellular stimuli [4,7,86,87]. RBPs can facilitate or inhibit miRNA processing either by recognizing and binding to RNA sequences or structures, or altering the function of the machinery involved in a specific step of processing (Figure 2).

#### 2.2.1. The Regulation of Pri-miRNA Processing

Altering the structure of pri-miRNAs to promote the binding or action of the Microprocessor is a common form of regulating pri-miRNA processing. For example, p72 (DDX17) is a DEAD-box helicase subunit of the Microprocessor [17,88,89] which facilitates pri-miRNA processing by binding to the 3’ flanking segments of the stem of the pri-miRNA hairpin [88,90,91]. While p72 is not required for the efficient production of all miRNAs, the production of a large subset of miRNAs are inhibited by the loss of p72 [88]. In addition, data generated by p72 CLIP experiments showed that 160 pri-miRNAs bind to p72, and most of these associations were mapped to the stem of the pri-miRNAs [90]. p68 (DDX5) is also a DEAD-box helicase subunit of the Microprocessor complex [17,89] that is required for the efficient processing of a specific subset of pre-miRNAs [92]. p68 recognizes the internal bulges of the pri-miRNA stem and unwinds them in an ATP-dependent manner, which promotes the binding of the Microprocessor complex [93].

RBPs can also regulate the processing of pri-miRNAs by recognizing and binding to the terminal loop of the pri-miRNAs [94]. For example, the heterogenous ribonucleoprotein A1 (hnRNPA1) is required for the efficient processing of pri-miR-18a, which is part of miR-17–92 cluster [95]. hnRNPA1 promotes pri-miR-18a processing by binding to its stem loop and generating a relaxed loop structure that is more accessible for the Microprocessor and enhances Drosha cleavage [95]. Conserved miRNA loops have been proposed to have the potential to bind other RBPs mainly from the hnRNP protein family that may also influence pri-miRNA processing [96].

The loop can also provide a platform for proteins that inhibit pri-miRNA processing. For instance, Lin28 negatively regulates *let-7a* biogenesis by binding to the pri-miRNA loop sequence which inhibits the effective association of the Microprocessor [97].

In addition to recognizing structures on pri-miRNAs, RBPs can influence pri-miRNA processing via binding to specific sequences. Interestingly, hnRNPA1, besides promoting miR-18 processing, can also have an inhibitory role in pri-miRNA processing. It binds to the GGG sequence of the pri-let-7a loop and displaces another RBP, KHSRP, a KH-type splicing protein, which is necessary for efficient pri-let-7a and pre-let-7a processing [94,98]. Another heterologous nuclear RNA-binding protein, hnRNPA2B1 binds to m^6^A sites (a site of methylation of the N^6^ nitrogen in adenosine, the most common internal modification of eukaryotic messenger RNA) located in the flanking regions of a subset of pri-miRNAs and facilitates the recruitments of the Microprocessor complex [99]. pri-miR-7 processing is also facilitated by direct binding of an RBP. In this case Serine/Arginine-Rich Splicing Factor 1 (SF2/ASF) recognises sequence motives on the miRNA stem region [100]. Fused in sarcoma (FUS) binds to the GU-rich elements of pri-miRNAs and promotes pri-miRNA processing of a distinct subset of miRNAs [101] (Figure 2a).

RBPs could also regulate pri-miRNA biogenesis by affecting the function of the Microprocessor without apparent binding to pri-miRNAs. Early proteomics study showed that members of the FET protein family, which is composed of FUS, EWS (Ewing Sarcoma protein) and TATA Box Binding Protein Associated Factor, (TAF15) proteins, co-immunoprecipitate with the Microprocessor [17] and subsequently all FET proteins have documented roles in the processing of miRNAs [102]. FUS controls the biogenesis of a subset of miRNAs by directly interacting with the Microprocessor and recruiting it to the transcription sites of miRNAs [101,103]. EWS affects miRNA biogenesis by inhibiting the expression of Drosha possibly by binding to its promoter region [104].

#### 2.2.2. The Regulation of Pre-miRNA Processing

Similar to pri-miRNA processing, the turnover and further maturation of pre-miRNAs are also extensively regulated by auxiliary RBPs. The majority of these RBPs bind to the pre-miRNA and either affect the stability of the RNA or modulate Dicer binding and/or function.

KHSRP regulates the processing of a subset of miRNAs by binding to G-rich regions of the terminal loops of their precursors [98]. This binding promotes Dicer association to the pre-miRNA and subsequently increases the cleavage rate of the precursors. TAR DNA-binding protein-43 (TDP-43) regulates the processing of two miRNAs, pre-miR-143 and 547, by binding to UG rich motifs in their respective terminal loops [105]. The cold-stress induced protein RBM3 regulates pre-miRNA production through binding pre-let-7 and pre-miR-16 directly and promoting their association with Dicer [106].

miRNA precursors can also be subjected to modifications and RNA nuclease activities that decrease their stability and result in impaired miRNA production. The most extensively studied mechanism that regulates miRNA turnover is the inhibition of miRNA processing mediated by Lin28. Lin28 binds to GGAG sequences in the terminal loop of the pre-miRNA of let-7, miR-107, miR-143 and miR-200c and recruits two Terminal Uridylyl Transferases (TUTases) that polyuridynylate the 3′ end of the pre-miRNA. This leads to the degradation of the precursor [107,108,109]. Currently, MCPIP1 (monocyte chemo attractant protein-MCP-induced Protein 1) is the only example of an endo-RNAse that regulates the stability of select pre-miRNAs by endonucleolytic cleavage [110]. MCPIP1 cleaves the terminal loops of the precursors which inhibit the binding of Dicer and accelerate the turnover of the pre-miRNAs [110]. Dicer recognizes and cleaves pre-miRNAs that are monophosphorylated at their 5′ ends [38,111,112]. The RNA-methyltransferase BCDIN3D interferes with the processing of pre-miR-145 by methylating its 5′ nucleotide that prevents Dicer binding and processing [113] (Figure 2b).

RBPs could also regulate miRNA biogenesis by affecting the expression or stability of the canonical proteins of the miRNA pathway. For example, the RBP AU-binding factor 1 (AUF1) regulates the general miRNA biosynthesis by inhibiting Dicer expression. AUF1 binds to the coding regions and the 3′ UTR of the Dicer mRNA and accelerates its turnover and decreases its expression [114].

#### 2.2.3. Regulation of the Turnover of Mature miRNAs

An increasing number of studies show that the turnover rate of mature miRNAs are not uniform suggesting the existence of regulatory mechanisms that regulate the stability of individual miRNAs [115,116,117]. The exact mechanism of this phenomenon has not been revealed yet but RBPs that degrade or modify mature miRNAs have been identified in a wide range of organisms [118].

In mammals 5′ to 3′ (XRN1) and 3′ to 5′ exonucleases (RRP41 and PNPase) have been demonstrated to degrade a subset of miRNAs [119,120]. QKI, a member of the signalling transduction and activation of RNA (STAR) family represents a unique regulatory mechanism in which QKI and its isoforms directly bind to miR-20 and stabilize it [121] (Figure 2c).

#### 2.2.4. RNA-Binding Proteins that Influence the Recognition and Regulation of miRNA Targets

**Figure 3:**
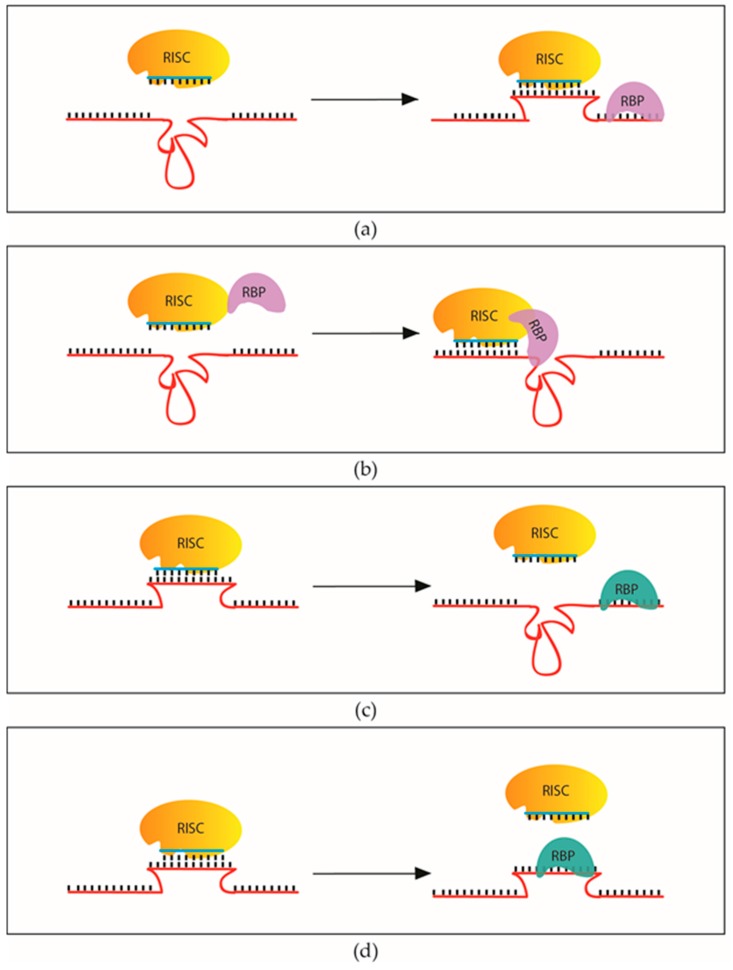
RNA binding proteins can promote and inhibit miRNA action. (**a**) Restructuring the target RNA by RBPs could result in miRNA targeting; (**b**) RBPs assisting miRNA targeting via direct binding to RNA induced silencing complex (RISC); (**c**) RBPs could restructure the target RNA and prevent miRNA targeting; (**d**) RNA binding proteins (RBPs) and miRNA complexes can compete to target the same mRNA via overlapping target sites.

In mammals, miRNAs mainly bind to the 3′ UTR of the target mRNAs [122]. The 3′ UTR is also a hotspot for RNA-binding proteins that regulate maturation, stability, transfer, localization and translation of mRNAs [123,124]. Therefore, it is inevitable that the miRNA loaded RISCs interact with other RBPs when numerous RBPs may bind to the same 3′ UTR. These interactions could result in the inhibition of miRNA action either by competing for the same binding motif or restructuring the RNA so that it becomes inaccessible for RISC complexes. On the other hand, RBPs could also change the structure of the 3′ UTRs to favour miRNA binding that facilitates miRNA mediated gene regulation. A particular RBP could be either an inhibitor or an enhancer of miRNA targeting depending on the context of its association with the mRNAs (Figure 3).

HuR (human antigen R) could regulate miRNA action through diverse mechanisms. For instance, HuR was shown to compete with a few miRNAs over potential binding sites and could inhibit miRNA targeting on select mRNAs (Figure 3d). It relieves the translational repression of TOP2A, CAT-1, COX-2 and ERBB2 by masking the miRNA target sites for miR-548c-3p, miR-122, miR-16 and miR-331 respectively [125,126,127]. HuR could also inhibit miRNA mediated gene regulation by preventing the RISC dependent dissociation of eIF4A from the translation initiation complex [85] (Figure 1d). On the other hand, HuR could also facilitate miRNA mediated gene regulation. It promotes let-7a binding by increasing binding site accessibility to the c-Myc 3′ UTR [128] (Figure 3a).

Polyprimidine tract binding protein (PTB) or hnRNP, an RBP with well described function in splicing and alternative splicing, also has dual functions in miRNA mediated gene regulation by promoting or inhibiting miRNA targeting through an RNA dependent interaction with the RISC [129].

DND1 also inhibits the targeting potential of multiple miRNAs. Binding to the U rich regions of target sites of miR-221 and miR-372, DND1 protects certain mRNAs from miRNA mediated repression [130]. Interestingly, DND1 function in regulating miRNA mediated gene expression is conserved since it regulates the accessibility of miRNAs in zebrafish to regulate early development [131].

In cancer cells, coding region determinant binding protein (CRD-BP) competes with miR-340 on the 3′ UTR of MITF and with miR-183 on the βTrCP1 transcript preventing their downregulation [132,133].

In addition, RNA Binding Motif Protein 38 (RBM38) inhibits miRNA targeting by binding to U-rich region in the proximity of miRNA target sites [127].

### 2.3. Transcriptome Wide Identification of RBPs that Modulate miRNA Mediated Gene Regulation

Cross-linking immunoprecipitation (CLIP) using Ago specific antibodies is being used to experimentally identify the Ago bound transcriptome that includes transcripts which are targeted by miRNAs [9,134]. This method has also been used to identify the footprint of different RBPs on whole transcriptomes [10,135,136,137,138,139,140]. Combining these approaches revealed that the interaction between miRNA targeting and RBP binding are much more widespread than it was originally thought.

Ago2 CLIP combined with PTB knock down experiments showed that PTB could inhibit or facilitate miRNA mediated gene regulation either to bind to miRNA target sites or restructure the 3′ UTR of the co-targeted mRNAs [141]. PAR-CLIP analysis of Pumilo (PUM) revealed that PUM sites co-localize 50 nt to the proximal region of specific miRNA sites and enhance miRNA action in human B cells [142]. CLIP based analysis also revealed the interactions of two RBPs, Moloney Leukemia Virus 10 (MOV10) and Fragile X Mental Retardation Protein (FMRP), in regulating miRNA mediated gene regulation. MOV10 was mapped to bind GC-rich sequences to facilitate miRNA targeting while FMRP could counteract this cooperativity by binding to or near MOV10 sites [143].

#### Comparison of Ago2 (Argonaute 2) and RBPs (RNA Binding Proteins) Binding in HeLA Cells Using Published CLIP Data

To investigate to what extent the binding site of AGO2 and other RBPs could overlap in a genomic scale, we conducted a preliminary bioinformatics analysis in which we compared the results of CLIP experiments carried out with a range of RBPs including Eukaryotic initiation factor 4AIII (eIF4AIII) [136], hnRNPC [144], PTB [141], T-cell intracellular antigen 1 (TIA1) [145], TIA1-like 1 (TIAL1) [145], U2 small nucleolar RNA auxiliary factor 65 (U2AF65) [144] and Upframeshift 1 UPF1 [140] in HeLa cells and compared these data generated with Ago2 CLIP data [141]. Our preliminary results show that a considerable number of RNAs identified by Ago2 CLIP overlap with sequences bind to at least one other RBP (Table 1).

**Table 1:** Identification of overlapping RBP and Ago2 binding sites

RBP	Number of Binding Sites	Number of Overlaps with Ago2 Binding Sites	Percentage of Overlap in Ago2
eIF4AIII	364,659	35,872	21%
hnRNPC	438,360	6422	4%
PTB	308,980	40,618	24%
TIA1	21,884	5029	3%
TIAL1	51,751	9029	5%
U2AF65	1,122,142	40,904	24%
UPF1	141,390	14,748	9%

The analysis of PAR-CLIP, HITS-CLIP and iCLIP datasets obtained from the StarBase version 2 [146,147] shows that ~50% of Ago2 interaction sites in HeLa cells co-localize with the binding site of at least one RBP (Table 1). Interestingly, we also found that the sequenced read numbers are significantly (*p* < 0.05) higher if they are covering sites that are recognized by Ago2 and other RBPs. This suggests that these sites are hotspots for RNA binding; therefore, inherently could be subjected to interactions such as competition and/or cooperation between different RBPs.

## 3. Materials and Methods

### Bioinformatics

CLIP-seq data (iCLIP, PAR-CLIP and HITS-CLIP) generated with AGO2 and multiple RBPs including eIF4AIII, hnRNPC, PTB, TIA1, TIAL1, U2AF65 and UPF1 in HeLa cells were downloaded from StarBase v2.0. The RBPs and Ago2 bound RNA fragments were mapped to human genome version hg19. RNA fragments that were bound to AGO2 as well as to any of the analysed RBPs with at least one nucleotide overlap were counted, and the corresponding sequencing read numbers were quantified. A significant calculation was carried out using one paired t-test in SPSS statistics tool.

## 4. Conclusions

RBPs are key proteins not only involved in generating miRNAs and carrying out their function but they also regulate miRNA processing and action at each step of the miRNA pathway. The identifications of RBP binding sites on mRNAs using next generation sequencing suggest that RBPs interaction with miRNA mediated gene regulation is potentially more widespread than the number of the verified interactions would suggest. Most of the RBPs that have documented roles in the regulation of the miRNA pathway act in a very specific environment. Therefore, it is highly likely that most of these predicted potential interactions are the manifestation of responses to cellular or environmental stimuli or specific to developmental stage or cell type.
